# Evaluation of Brain Death in Laying Hens During On-Farm Killing by Cervical Dislocation Methods or Pentobarbital Sodium Injection

**DOI:** 10.3389/fvets.2019.00297

**Published:** 2019-09-03

**Authors:** Elein Hernandez, Fiona James, Stephanie Torrey, Tina Widowski, Karen Schwean-Lardner, Gabrielle Monteith, Patricia V. Turner

**Affiliations:** ^1^Department of Pathobiology, University of Guelph, Guelph, ON, Canada; ^2^Department of Clinical Studies, University of Guelph, Guelph, ON, Canada; ^3^Department of Animal Biosciences, University of Guelph, Guelph, ON, Canada; ^4^College of Agricultural and Bioresources, University of Saskatchewan, Saskatoon, SK, Canada

**Keywords:** animal welfare, EEG, chicken, poultry, euthanasia, cervical dislocation, pentobarbital sodium

## Abstract

This study investigated changes in the electroencephalograph (EEG) power spectrum as well as physiological and behavioral responses to on-farm killing via mechanical cervical dislocation (MCD), manual cervical dislocation (CD) or intravenous pentobarbital sodium administration in lightly anesthetized laying hens, to evaluate the welfare impact of each method. A mixed group of 44 white Leghorn and Smoky Joe laying hens (60 weeks-old) were anesthetized with isoflurane in oxygen and maintained at 1.5–2% isoflurane/O_2_ until the killing method was applied. Birds were randomly assigned to one of three experimental groups on each trial day. The EEG was recorded bilaterally in a four-electrode montage. After recording a 5-min baseline, the killing method was applied and EEGs and other behavioral and physiological responses, including convulsions, gasping, cessation of body movements and feather erection were recorded for 5 min. Changes in EEG frequency bands (alpha, beta, delta, theta), median frequency (F50), 95% spectral edge frequency (F95), and total power (Ptot) were used to assess the quality of the on-farm killing event. Within 15 s after administration of pentobarbital sodium, there were significant decreases in mean frequency bands, increases in mean F50 and F95, and decreases in Ptot, suggesting brain death. In addition, birds presented a shorter latency to cessation of movement after pentobarbital sodium injection compared to MCD and CD (22 vs. 115 s and 136 s, respectively). There were significant increases in F95 and decreases in Ptot at 120 s after application of CD; and a concomitant decrease in the frequency bands at 135 s and isoelectric EEG at 171 ± 15 s. Changes consistent with brain death after MCD included isoelectric EEG at 207 ± 23 s and a significant decreases in some frequency bands at 300 s post-application. No other significant spectrum frequency changes were observed in the MCD group, suggesting brain death likely occurred near the 5-min endpoint. There was no clear association between behavioral, physiological, and EEG responses within CD and MCD treatments. The data demonstrate that pentobarbital sodium induced a rapid death with minimal behavioral and physiological responses regardless of strain of hens. In comparison, use of CD and MCD resulted in a slow onset of brain death in hens.

## Background

Culling birds on-farm is required periodically, and poultry producers and farm personnel working with birds must ensure that these animals are killed promptly and humanely. Cervical dislocation is a conditionally accepted method of on-farm killing in the poultry industry and can be applied either manually or mechanically (1). From an animal welfare perspective, euthanasia methods should minimize potential pain and distress and result in rapid loss of sensibility without recovery (2). However, there are few reports about the welfare impact of different cervical dislocation methods in laying hens (3–5).

Manual cervical dislocation (CD) is the most common on-farm killing method for laying hens, but there are several welfare concerns associated with CD, such as prolonged time to insensibility, incorrect site of application, and observer esthetics (4, 6, 7). When CD is correctly applied, the neck dislocation must separate the cervical vertebrae and sever the spinal cord and carotid arteries, ideally between the skull and the first cervical vertebra (2, 8, 9). Brain death is the result of hypoxia due to reduced blood flow (8). Because of potential difficulties encountered with manual CD related to bird weight and operator strength and fatigue (1, 10), different mechanical cervical dislocation (MCD) methods have been developed (4, 7, 11, 12). However, there are multiple device designs with different efficacy rates (13). For example, pliers and some other MCD devices do not always sever the spinal cord or major cervical blood vessels (7–9). In addition, injuries secondary to the application of different MCD tools are potentially painful, and the onset of insensibility may be challenging to assess (6, 8, 9). The Koechner Euthanizing Device® (KED) is a relatively novel tool designed for poultry on-farm killing (14). A recent study demonstrated a longer time to brain death with KED compared to CD in broilers (15), but there is limited information in laying hens (5). Due to the lack of information on the efficiency and humanness of this method the term “on-farm killing” and not euthanasia was used in the present study for MCD and CD. In contrast to CD and MCD, the American Veterinary Medical Association (AVMA) and the Canadian Code of Practice for laying hens consider intravenous (IV) barbiturate overdose to be the gold standard method of euthanasia for most species and is considered as an appropriate method approved without conditions for poultry (1, 2). Pentobarbital sodium is a barbituric acid derivative that causes short-acting, dose-dependent depression of the central nervous system. However, its use is restricted to licensed veterinarians, and carcasses cannot be rendered or used for other purposes (1, 2).

The assessment of insensibility and death is complex and requires the use of more than one indicator (16). Study of electroencephalogram (EEG) is one objective means of determining cerebral cortical function, and the technique has been used in small animal veterinary medicine for anesthesia monitoring and other forms of neurologic investigation (17, 18). Other indirect indicators of brain activity associated with sensibility are brainstem and spinal reflexes, including corneal, pupillary light, nictitating membrane, and pedal reflexes have been reported in poultry (3, 15, 16, 19–21). Loss of these reflexes occurs during different anesthesia planes and can indicate insensibility in chickens (19). In addition, other measures can be used to assess insensibility and death, such as neck muscle tone, convulsions, feather erection, and heart and respiratory rates in chickens and turkeys (7, 12, 16, 22).

Several researchers have employed EEG to determine sensibility and death in poultry undergoing different euthanasia and on-farm killing methods (3, 23, 24). In general, insensibility under anesthesia is observed as a synchronized EEG activity characterized by a shift toward the low frequencies of the EEG spectrum (delta (<4 Hz) and theta (4–8 Hz) frequency bands) with an increase in the amplitude of the EEG signal (19, 25). Sandercock et al. (19) described shifts from sensibility to insensibility as decreases in median (F50) and spectral edge (F95) frequencies with a sharp increase in the total spectral power (Ptot) in chickens and turkeys. Ptot is the absolute power recorded in the EEG spectrum (26), and it is at its lowest value when birds are dead, reflecting an isoelectric EEG or cerebral inactivity (27–29). F50 and F95 are derived from the EEG power spectrum and represent the frequency below which 50% and 95% of the total power is located (25, 26).

The objective of this study was to assess the welfare impact of two cervical dislocation methods for on-farm killing of two strains of laying hens in comparison with intravenous pentobarbital sodium using EEG, behavioral and physiological measurements. Because MCD is a novel device with an unknown outcome, the study was conducted in anesthetized birds using isoflurane to minimize any potential pain or distress associated with the method. The results of each euthanasia or killing method were analyzed in three parts: (i) the analysis of different quantitative EEG variables in response to the method, (ii) the study of latency to behavioral and physiological measures, and (iii) the relationship between the EEG, behavioral, and physiological responses. We hypothesized that there would be a different time to brain death between the physical and chemical killing methods.

## Methods

### General Description of Subjects and Experimental Design

In total, 44 hens (60 week-old [w.o.]) of two different strains (white Leghorn and Smoky Joe) were sourced from the University of Guelph flocks (Table 1) based on availability at the time of the experiments. All hens were selected for euthanasia or on-farm killing since they were at the end of their experimental utility at the research station. All experiments were approved by the University of Guelph Animal Care Committee (AUP 3321) and birds were randomly allocated to one of the three on-farm killing treatments on trial days: barbiturate overdose (*n* = 3 white Leghorn; *n* = 5 Smoky Joe), CD (*n* = 8 white Leghorn; *n* = 12 Smoky Joe), and MCD (*n* = 4 white Leghorn; *n* = 12 Smoky Joe). The methods were randomized by day using a random number generator (random.org). Preparation time, application of the method, and recording time for each bird took ~30–40 min. Birds were killed over five trials, with no more than 11 birds tested with either cervical dislocation method each day and 2 or 3 birds euthanized with pentobarbital sodium overdose per day. These numbers were estimated to avoid operator burnout and to conduct the experiments during the working hours of the farm. The cervical methods were balanced for the Smoky Joe strain. The experiments with Leghorn hens were conducted first over the first 2 days of the study followed by the Smoky Joe hens due to the availability of birds at the farm at the time of experiments. For consistency, a single experienced operator performed each of the CD and MCD applications. A licensed veterinarian administered the barbiturate (pentobarbital sodium) according to Canadian guidelines (1).

**Table 1:** Distribution of treatment groups by age, weight, and euthanasia method with the sample size indicated for each group of white Leghorn and Smoky Joe hens.

**Strain**	**Weight (kg) ± SE**	**Method**	**Sample size**
White leghorn	1.9 ± 0.15	Pentobarbital sodium	3
White leghorn	1.5 ± 0.02	CD	8
White leghorn	2 ± 0.04	MCD	4
Smoky joe	1.7 ± 0.09	Pentobarbital sodium	5
Smoky joe	1.6 ± 0.07	CD	12
Smoky joe	1.7 ± 0.02	MCD	12

**Figure 1:**
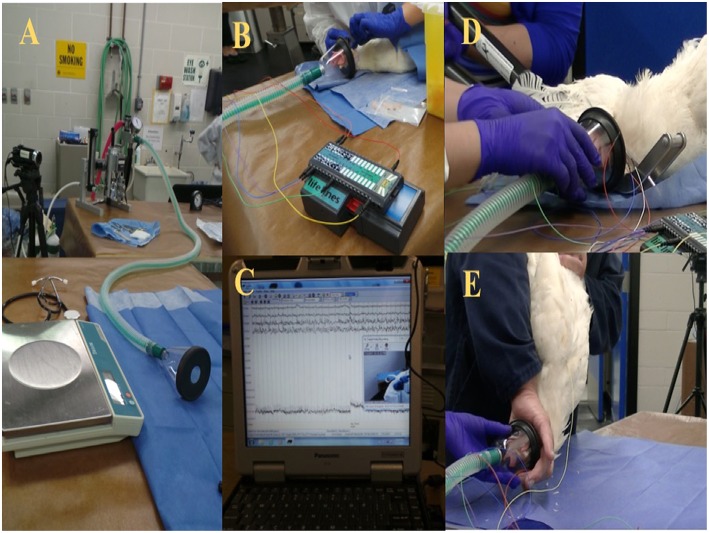
**(A)** Experimental set up preparation table with scale and isoflurane anesthesia machine. **(B)** Wireless telemetry system and subcutaneous electrode placement. **(C)** Raw EEG recording. **(D)** Mechanical cervical dislocation application. **(E)** Manual cervical dislocation application.

The birds were brought from the barn as a group to the procedure room and were kept in a transportation crate for a period of 2–4 h for habituation. Isolation stress may have affected the last bird of each trial day. However, the time that the last bird was alone was <40 min. Birds were individually weighed and induced for anesthesia via a small facemask with 5% isoflurane (Isoflo, Abbot Laboratories, IL, USA) in oxygen (Figure 1). Subsequently, isoflurane anesthesia concentration was lowered to a maintenance/minimum anesthesia level of 2% for electrode placement and reduced again to 1.5% for ~5 min prior to the euthanasia or on-farm killing method being applied. The oxygen and the inhalant anesthesia were turned off immediately after the method application. All birds were truly euthanized, since all the experiments were conducted using light anesthesia to minimize or eliminate any potential pain or distress. However, the term euthanasia was only used for the anesthetic overdose because it is considered the gold standard euthanasia method. After the application of the euthanasia or on-farm killing method, birds were evaluated for 5 min and monitored for EEG activity and behavioral changes.

Operator technical efficacy was assessed with pilot studies using cadavers and anesthetized hens (data not shown) due to potential welfare concerns related to the mechanical method (MCD) that had been reported in previous studies in poultry (5, 15, 21). Radiographic evidence confirmed the site of CD and MCD application between the skull and C1 or C1-C2 and C1-C2 or C2-C3, respectively (5). Humane endpoints included the inability to collect a valid EEG signal, prolonged labored breathing, and inability to detect cervical damage through palpation immediately after the application of the methods. When any of these conditions were detected, a second euthanasia method was applied (Zephyr-EXL, Bock Industries Inc. Philipsburg, PA, USA).

## Data collection

### Implantation of EEG Electrodes and Baseline Recording

Each bird was placed in lateral recumbency and provided with 2% isoflurane/O_2_ for electrode placement after induction of anesthesia. Leg tone (i.e., tension in lifting the leg) and toe pinch (i.e., withdrawal of the foot in response to pressure to the toes) were continuously checked for anesthesia depth monitoring (3). Four 25-gauge needle EEG subdermal wire electrodes (Ives EEG Solutions, MA, USA) were placed as described by Eberle et al. (30) and James et al. (17), but modified for our purpose. The electrodes were placed 0.5 cm apart from the midline and over each hemisphere of the brain. The first pair of electrodes was placed on the frontal bone and the second pair on the parietal bone. A reference electrode was inserted on the anterior portion of the frontal bone (underneath the comb), and a neutral electrode was inserted at the base of the occipital bone. No movements were observed secondary to the electrode placement. All electrode tips were secured onto the head with surgical tape. The wire leads from the telemetry system (Trackit MK3 EEG and video recorder, Lifelines Neurodiagnostic Systems, IL, USA) were also secured to the facemask and rebreathing tube with surgical tape. A baseline EEG signal was tested for impedance and signal quality, and then proceeded with baseline recording for ~5 min at 1.5% isoflurane concentration to ensure a light anesthesia level based on pilot study work on anesthesia monitoring (data are not shown) and research on chicken anesthesia with isoflurane (31). The birds remained under anesthesia for a maximum of 7 min, including the time to electrode placement, and baseline recording. A 60 Hz notch filter was applied during EEG recording. The impedance was kept under 16 kΩ and recorded before the baseline started and after the post-euthanasia or killing recording was completed. EEG recording was monitored in real time during trials to ensure correct placement of probes and functioning of equipment, and the EEG recordings were saved using the Trackit MK3 software and onto an external hard drive for further analyses.

### Euthanasia or On-Farm Killing Methods and Procedure

Following the 5-min baseline recording, leg tone and toe pinch were evaluated to confirm insensibility, and the method was applied. The isoflurane and oxygen were turned off immediately after the method was applied. Each bird's head remained partially inside the facemask (e.g., beak and eyes and a cranial portion of the comb) and was not removed to minimize interference with the EEG signal. The barbiturate overdose was administered IV into the brachial vein (0.5 mL/kg) (Euthansol, Intervet Canada CORP, Merck Animal Health, QC, CA). The MCD device used was the KED (U.S. Patent Number 8,152,605) (14). The device is a modified non-penetrating forceps (model C, size medium) designed for poultry up to 13.6 kg that consists of a scissor-type mechanism with a 69 cm handle (14). Birds were positioned in sternal recumbency, and the neck was gently extended to place the blunt blades caudal to the occipital bone. The double-sided blade was placed ventrally, and the single blade was positioned dorsally to the animal's neck. The handles were closed together until the blades overlapped to cause transection of the spinal cord, according to the manufacturer's instructions (14). The CD method was performed by an experienced operator in compliance with the Canadian animal care guidelines for CD in laying hens (1, 32). The neck was dislocated by stretching the neck downward with one hand and the other hand restraining the legs and wingtips. The neck and the back of the head (i.e., the portion outside the facemask) were restrained with the index and middle finger while the rest of the head and facemask were held in the operator's hand. After the neck dislocation was conducted, all birds were immediately (<7 s) placed in sternal position on a table until the 5 min EEG and video recordings were complete. Occasionally, some birds killed with CD and MCD were manually restrained by gently holding the wings or legs onto the table to minimize any extracranial electrical noise source affecting the EEG signal from the body movements and for the operator's safety during the clonic convulsion phase. Eight birds in the MCD group were physically restrained to test a purpose-made fabric device to minimize convulsions. All birds on-farm killed with cervical dislocation, either CD or MCD, received a second euthanasia application method using a non-penetrating captive bolt device (Zephyr-EXL, Bock Industries Inc. Philipsburg, PA, USA) to ensure brain death after the experimental endpoint was reached.

### Behavioral and Physiological Responses Recording and Scoring

Two high definition video cameras (JVC GZ-E200 full HD Everio Camcorder, Yokohama, Japan) were mounted on tripods to record responses from the front and rear end of each bird. All videos clips were edited to remove the euthanasia or on-farm killing method, permitting blinded evaluation of responses to the method, followed by randomization of clips using a random number generator (random.org). Video recordings were initiated during electrode implantation and anesthesia maintenance and stopped 5 min after treatment application. Behavioral and physiological responses of each bird were scored continuously by a single trained observer blinded to the treatment using Observer XT (Version 9.0: Noldus Information Technology, Wageningen, Netherlands) according to an ethogram designed for this study (Table 2). However, the complete blinded evaluation was mildly affected because not all the responses were observed with all the applied methods, such as lack of clonic convulsions and gasping after pentobarbital sodium injection. The intraclass correlation coefficient (ICC) was determined to ensure behavioral and physiological responses were being scored consistently over time, and no drift had occurred (all intra-observer reliability tests produced an ICC above 0.8). Time to onset of clonic convulsions was subsequently scored from original videos for accuracy because convulsions often started immediately after application of the method and were sometimes edited out during video editing if the method was visible in the frame. Values are presented as mean ± SE of results from individual birds in each treatment.

**Table 2:** Description of parameters measured using video recordings after treatment application.

**Behavior**	**Description**
Gasping	Repetitive and rapid opening and closing of the beak (not associated with respiratory function)
Clonic convulsions	Rapid, uncoordinated movement of the body and wings
Tonic convulsions	Slow extension or movement of the legs and wings
Motionless	No body or respiratory movement
Feather erection	Multifocal to generalized rising of feather of the body
End time of last body movement	Time of last wing, leg or body movement or onset of motionless bird with no further movement

*Latencies were recorded for each variable. Adapted from Martin. et al. (33)*.

### Electroencephalographic Assessment

EEG data were analyzed after the recordings were completed. All EEG recordings were randomized using a random number generator (www.random.org) and exported to the Insight II software (Persyst Development Corporation, Prescott, AZ), where it was arranged in a channel-reference montage and carefully inspected for signal artifact and muscle activity prior to Fast Fourier Transformation. All hens acted as their own control. Twenty-four, artifact-free, 1 s epochs were manually selected by a single reviewer blinded to treatment at 15 s intervals post-euthanasia or killing (20 1 s epochs obtained in a 5 min recording post-euthanasia or killing and 4 1 s epochs during baseline recording) for each bird. A 15 s interval was used to collect 4 noise-free 1 s epoch per minute (34). The intraclass correlation coefficient (ICC) was determined to assess accuracy during epoch selection prior to fast Fourier transformation (all intra-observer reliability tests produced an ICC above 0.8 in scored EEG recordings).

EEG spectra were calculated using a Hamming window, with 256 points/window and 50% overlapping windows (35). The Fast Fourier Transformation was applied to the selected epochs to perform quantitative EEG (QEEG) analysis of frequency bands and other spectral variables. The brain waves or frequency bands were classified according to their approximate spectral boundaries in delta (1–4 Hz), theta (4–8 Hz), alpha (8–13 Hz), and beta (13–30 Hz). The other power spectrum derived variables included median frequency (F50), spectral edge frequency (F95), and total power. A high-frequency (60 Hz) notch filter was applied to eliminate noise based on Nevarez et al. (35). Brain death was determined on significant changes in EEG spectrum variables compared to baseline and the presence of isoelectric EEG (28). Isoelectric values in the EEG were determined by a single reviewer blinded as to treatment, visual scoring of decreased wave amplitude (36) with an isoelectric or flat line in a non-artifactual signal (≤2 uVpp) (28, 29).

### Statistical Analyses

Statistical analyses were carried out using SAS 9.4 (SAS Institute Inc., Cary, NC). A Fisher's exact test was used to test the null hypothesis that the proportion of chickens presenting with behavioral and physiological responses and isoelectric EEG was independent of the euthanasia/ killing method. Frequency tables were used to determine the numbers of hens presenting with each response. A mixed model analysis was conducted to test the fixed effects of the euthanasia/ killing method, strain, body weight and their interaction on the latency of clonic convulsions, tonic convulsions, time to last movement, gasping, feather erection, and isoelectric EEG of birds presenting such responses. Differences among least squares means were compared using a Tukey–Kramer test with significant effects determined at *p* ≤ 0.05. Residuals plots were examined to check deviations from normality. The method × strain and method × body weight interactions were first tested and removed from the model because the results showed no significant interactions. For the latency of clonic convulsions, tonic convulsions, time to last movement, gasping, feather erection, and isoelectric EEG of birds distribution was normal and the log transformation function was not used. The data were pooled for both strains to test the method and dependent value interaction. Correlations between latency to isoelectric EEG and latency to behavioral and physiological responses of birds were tested with a Pearson's correlation test. For all variables, least square means ± SE are reported and values of p ≤0.05 were considered significant.

A general linear repeated measures mixed model was used to test for mean differences in EEG parameters measured over time. The fixed effects of euthanasia or killing method, strain of bird, and time; as well as body weight and the quadratic of body weight were included in the model. The full model included all main effects and interaction terms. The models were simplified with non-significant effects removed. Akaike information criteria (AIC) lowest is best was used to determine which correlation structure to use to account for repeated measures. Dunnett's *post-hoc* test was performed for multiple comparisons between post-euthanasia or kill blocks and baseline. Tukey adjustment was used for multiple comparisons between methods or strains. Normality of data was tested using Shapiro-Wilk test, Kolmogorov-Smirnov test, and examination of the residuals. The parameter theta was log transformed to improve normality. Significance was set at *p* < 0.05. For all variables, raw means ± SE are reported.

## Results

Video and EEG recordings were successfully collected from the majority of the instrumented birds with the exception of three videos of the CD method and one EEG from the MCD group in the Smoky Joe group that were not saved due to technical issues (EEG recordings were successfully collected in 43 of 44 birds; video recordings were successfully collected in 41 of 44 birds). Remaining data sets were complete.

### Behavioral and Physiological Responses

The incidence of various behavioral and physiological parameters is presented in Table 3. There was a higher number of Smoky Joe chickens that presented clonic and tonic convulsions after on-farm killing by CD and MCD compared to pentobarbital sodium (clonic: 100% compared to 0%, *p* = 0.0005; tonic: 78% compared to 40%, *p* = 0.3; and clonic: 91% compared to 0%, *p* = 0.001; tonic: 58% compared to 40%, *p* = 0.6, respectively). Similarly, more white Leghorn hens exhibited clonic convulsions after MCD and CD compared with pentobarbital (100% compared to 0%, *p* = 0.03; 13% compared to 0%, *p* > 0.5). In contrast, tonic convulsions were observed in fewer white Leghorn chickens after MCD and CD compared to pentobarbital sodium administration (0% compared to 100%, *p* = 0.03; 75% compared to 100%, *p* = 0.5, respectively). There was no method effect on the number of birds that exhibited feather erection after either CD or MCD, regardless of the strain, compared to pentobarbital (Table 4). However, there was a method effect on the frequency of gasping in Smoky Joe and white Leghorn hens following MCD application compared to pentobarbital sodium (83% compared to 0%, *p* = 0.003; 100% compared to 0%, *p* = 0.03, respectively). Similarly, more Smoky Joe and white Leghorn hens presented with gasping after CD than those euthanized with pentobarbital sodium (56% compared to 0%, *p* = 0.09; 88% compared to 0%, *p* = 0.02) (Table 3).

**Table 3:** Absolute incidence (and percentage) of White Leghorn and Smoky Joe hens presenting with various behavioral and physiological parameters following euthanasia method application.

**Parameter**	**Smoky Joes**			**White leghorn**		
	**Pentobarbital sodium (*n* = 5)**	CD (*n* = 9)^*^	**MCD (*n* = 12)**	**Pentobarbital sodium (*n* = 3)**	**CD (*n* = 8)**	**MCD (*n* = 4)**
Clonic convulsions	0^a^ (0)	9^b^ (100)	11^b^ (91)	0^a^ (0)	1^a^ (13)	4^b^ (100)
Tonic convulsions	2^a^ (37)	7^a^ (78)	7^a^ (58)	3^a^ (100)	6^a^ (75)	0^b^ (0)
Feather erection	3^a^ (60)	8^a^ (89)	8^a^ (67)	3^a^ (100)	7^a^ (88)	3^a^ (75)
Gasping	0^a^ (0)	5^a^ (56)	10^b^ (83)	0^a^ (0)	7^b^ (88)	4^b^ (100)

* *Three video recordings were not available for behavioral scoring*.

a, b *Means within a row with different superscripts differ significantly at p ≤ 0.05*.

**Table 4:** Least square means ± SE with minimum and maximum data of latency of physiological and behavioral parameters following euthanasia method application in white Leghorn and Smoky Joe hens.

**Parameters**	**Method**									**F**	***p-*value**
	**Pentobarbital sodium**			**CD**			**MCD**				
	**Mean ± SE**	**Min**.	**Max**.	**Mean ± SE**	**Min**.	**Max**.	**Mean ± SE**	**Min**.	**Max**.		
Clonic convulsions	–	–	–	2 ± 0.2^a^	0.3	7	0.3 ± 0.2^b^	0.1	5	18.82	0.0007
Tonic convulsions	35 ± 33^a^	12	56	130 ± 20^b^	3	256	187 ± 27^b^	136	262	6.4	0.007
Time to last movement	47 ± 38	0.8	94	154 ± 26	1.5	318	106 ± 27	0.2	314	2.8	0.07
Gasping	–	–	–	55 ± 12^a^	19	97	110 ± 13^b^	9	245	8	0.0009
Feather erection	22 ± 26^a^	1	39	151 ± 16^b^	57	245	136 ± 19^b^	5	252	9.3	0.0008
Isoelectric point	20 ± 23^a^	10	39	171 ± 15^b^	81	317	207 ± 23^b^	31	289	19.6	<0.0001

a, b *Means within a row with different superscripts differ significantly at p ≤ 0.05*.

*Groups that did not present the responses were scored as missing values (–)*.

*Minimum and maximum data are from raw data*.

Clonic convulsions were characterized by aggressive wing flapping and overall body movement usually followed by tonic convulsions (Table 4). The onset of clonic convulsions was within the first few seconds after CD and MCD application with a shorter latency after MCD compared with CD (0.3 ± 0.2 s compared to 2 ± 0.2 s; *p* = 0.0007, respectively). There was a significant method effect observed on the latency to tonic phase with prolonged latency in MCD compared to CD and pentobarbital sodium euthanasia groups (187 ± 27 s compared to 130 ± 20 s and 35 ± 33 s; *p* = 0.007, respectively). There was no method effect on the latency to the last body movement between treatment groups (*p* = 0.07); however, CD and MCD had a tendency to a longer time of onset to last body movement compared to pentobarbital euthanasia treatment (154 ± 26 and 106 ± 27 s compared to 47 ± 38 s, respectively) (Table 4). Birds killed with CD had a shorter latency to gasping compared to birds on-farm killed with MCD (55 ± 12 s compared to 100 ± 11 s; *p* = 0.01). No birds euthanized with pentobarbital sodium exhibited gasping. Feather erection had a shorter mean latency after pentobarbital sodium compared to both physical methods (*p* = 0.0008).

### EEG Responses

The number and percentage of EEG epoch collected for evaluation and successfully used are described in Table 5. There was a significant effect of strain × time interaction in alpha, beta, theta, and total power (Supplementary Table 1). Similarly, there was a significant effect of strain × time × treatment interaction in F50 and F95 (Supplementary Table 2). The significant strain × time interactions were observed at baseline in all frequency bands and Ptot (adjusted *p* ≤ 0.05) and at 45 s between both strains with alpha and beta (adjusted *p* ≤ 0.05). The significant strain × time × treatment interactions in F50 were observed at 60 s after pentobarbital sodium (adjusted *p* = 0.04) and at baseline in CD (adjusted *p* = 0.02) between strains. F95 showed a significant strain × time × treatment interaction at 105 and 120 s (adjusted *p* = 0.04). The strain effect and strain associated interactions were removed from the final model since it was likely caused by artifact noise during the experiment day. Statistical analyses revealed no effect of euthanasia/ killing method × time interaction on all frequency bands and total power (Tables 6, 7). There was a significant method × time interaction on F50 and F95 (Table 7). There was a method and time effect on all EEG variables (Tables 6, 7). The incidence of hens presenting with an isoelectric EEG is presented in Table 4. Means and standard errors for all spectrum variables during baseline and post-method application time points are listed in Tables 8–**10**.

**Table 5:** Number of epochs evaluated for quantitative EEG analysis.

**Euthanasia method**	**Strain**	**No. of birds**	**No. of epochs evaluated for EEG analysis per bird**	**No. of epochs selected for baseline EEG analysis**	**No. of epochs selected for post-euthanasia EEG analysis**	**Total number of used epochs (%)**
Pentobarbital sodium	white leghorn	4	72	12	78	57 (79%)
	Smoky joe	11	120	20	212	117 (98%)
Manual cervical dislocation	White leghorn	8	132	32	143	175 (91%)
	Smoky joe	12	288	48	229	277 (96%)
Mechanical cervical dislocation	White leghorn	4	96	16	45	94 (91%)
	Smoky joe	11	264	44	97	256 (96%)

**Table 6:** Final summary effects of euthanasia method on EEG frequency bands in white Leghorn and Smoky Joe hens.

	**Delta**		**Theta**		**Alpha**		**Beta**	
**Effect**	***F***	**Pr > *F***	***F***	**Pr > *F***	***F***	**Pr > *F***	***F***	**Pr > *F***
Method	7.8	0.003	7.6	0.0007	6	0.003	11.5	<0.0001
Time	2	0.008	3.6	<0.0001	0.8	0.8	2.2	0.0009
Time × method	1.2	0.2	1.1	0.3	1.0	0.7	0.7	0.9
Body weight	0.8	0.4	3.4	0.07	1.6	0.2	0.11	0.8
Body weight × method	5.1	0.007	8.29	0.0004	6.8	0.0015	11.7	<0.0001

*Only significant interactions are included*.

**Table 7:** Final summary effects of euthanasia method on EEG spectral frequencies and Ptot in white Leghorn and Smoky Joe hens.

	**F50**		**F95**		**PTOT**	
**Effect**	***F***	**Pr > *F***	***F***	**Pr > *F***	***F***	**Pr > *F***
Method	2.1	0.1	0.4	0.7	5.2	0.04
Time	2.4	0.0007	6	<0.0001	2.5	0.001
Method × time	3.3	<0.0001	2.5	<0.0001	0.7	0.7
Body weight	4.3	0.04	6.7	0.01	2.5	0.0001
Body weight × method	2.1	0.13	0.4	0.01	2.7	–
Body weight × body weight	–	–	–	–	–	0.0001

**Table 8:** Mean EEG parameters (SE) after administration of pentobarbital sodium.

**Time (s)**	**Delta (μV)**	**Theta (μV)**	**Alpha (μV)**	**Beta (μV)**	**F50 (Hz)**	**F95 (Hz)**	**PTOT (μV^**2**^)**	N epochs^**^
	**Mean (SE)**	**Mean (SE)**	**Mean (SE)**	**Mean (SE)**	**Mean (SE)**	**Mean (SE)**	**Mean (SE)**	
Baseline	2.6 (0.5)	3.4 (0.7)	2.2 (0.5)	1.1 (0.3)	4.7 (0.3)	14.0 (1.1)	36.6 (9.2)	32
15	0.9 (1.3)	1.2 (1.0)	0.9 (0.4)*	0.8 (0.4)	6.5 (0.5)*	23.0 (1.8)*	5.5 (9.2)	8
30	0.4 (0.5)	0.6 (0.5)*	0.5 (0.3)*	0.7 (0.2)	8.5 (0.7)*	24.8 (1.6)*	2.0 (9.2)	8
45	0.5 (0.5)*	0.6 (0.4)*	0.5 (0.2)*	0.6 (0.2)	9.5 (0.7)*	24.8 (2.1)*	1.6 (9.2)	8
60	0.5 (0.5)*	0.5 (0.4)*	0.4 (0.2)*	0.5 (0.1)	6.5 (0.4)*	24.3 (2.3)*	1.2 (9.2)	8
75	0.3 (1.1)	0.4 (0.6)*	0.4 (0.4)	0.5 (0.3)	9.5 (0.6)*	25.0 (1.9)*	1.0 (9.2)	8
90	0.3 (1.0)	0.3 (0.7)*	0.3 (0.3)*	0.3 (0.3)	7.0 (0.6)*	24.0 (1.9)*	0.4 (9.2)	8
105	0.3 (0.8)	0.3 (0.6)*	0.2 (0.3)*	0.3 (0.3)	6.3 (0.6)	25.5 (1.9)*	0.4 (9.2)	8
120	0.3 (1.0)	0.4 (0.6)*	0.3 (0.3)*	0.3 (0.2)	6.5 (0.6)	24.0 (2.0)*	0.5 (9.2)	8
135	0.3 (1.0)	0.3 (1.0)	0.3 (0.3)*	0.3 (0.2)	7.5 (0.9)	24.8 (2.3)*	0.3 (9.2)	8
150	0.3 (0.9)	0.3(0.5)*	0.3 (0.3)*	0.4 (0.2)	8.5 (0.9)*	25.5 (2.1)*	0.6 (9.2)	8
165	0.4 (0.7)	0.4 (0.5)*	0.3 (0.3)*	0.3 (0.2)	7.5 (1.2)	24.8 (2.1)*	0.7 (9.2)	8
180	0.4 (0.3)*	0.4 (0.2)*	0.3 (0.3)*	0.3 (0.3)	6.3 (1.2)	24.5 (1.9)*	0.5 (9.2)	8
195	0.4 (0.6)	0.4 (0.3)*	0.3 (0.1)*	0.3 (0.1)	6.9 (0.8)	23.4 (1.8)*	0.5 (9.8)	7
210	0.4 (0.6)	0.4 (0.2)*	0.3 (0.1)*	0.3 (0.1)	6.6 (1.0)	22.8 (2.0)*	0.5 (9.8)	7
225	0.5 (0.3)*	0.5 (0.3)*	0.3 (0.1)*	0.3 (0.1)	5.4 (1.1)	23.1 (1.9)*	0.7 (9.8)	7
240	0.4 (0.7)	0.4 (0.3)*	0.2 (0.1)*	0.3 (0.1)	6.6 (1.2)	24.1 (2.0)*	0.5 (10.6)	6
255	0.6 (0.3)*	0.6 (0.3)*	0.3 (0.1)*	0.3 (0.1)	4.6 (0.9)	21.4 (2.2)*	1.1 (10.6)	6
270	0.4 (0.3)*	0.5 (0.3)*	0.3 (0.1)*	0.4 (0.1)	5.7 (0.9)	23.4 (1.9)*	0.7 (10.6)	6
285	0.4 (0.5)*	0.4 (0.3)*	0.3 (0.2)*	0.3 (0.1)	5.8 (1.2)	24.9 (2.5)*	0.4 (12.9)	4
300	0.5 (0.6)	0.4 (0.4)*	0.3 (0.2)*	0.4 (0.1)	5.6 (1.6)	23.4 (2.8)*	0.6 (15.0)	3

Comparison of the mean and standard error (SE) of the frequency band powers (delta, theta, alpha, beta), spectral frequencies [median frequency (F50) and spectral edge frequency (F95)] and absolute power (PTOT) of the avian electroencephalogram at consecutive 15 s intervals after administering pentobarbital sodium in laying hens. Data are shown as mean values over consecutive 15 s intervals after euthanasia, with baseline representing the mean of the 60 s immediately euthanasia. Asterisks indicate values within columns that are significantly different from baseline (adjusted p ≤ 0.05).

** *Number of analyzed epochs varies as not every epoch per 15 s interval was available for every bird. Baseline parameter are based on 4 epoch per bird, post-euthanasia parameters are based on 20 epochs obtained in a 5 min recording*.

**Figure 2:**
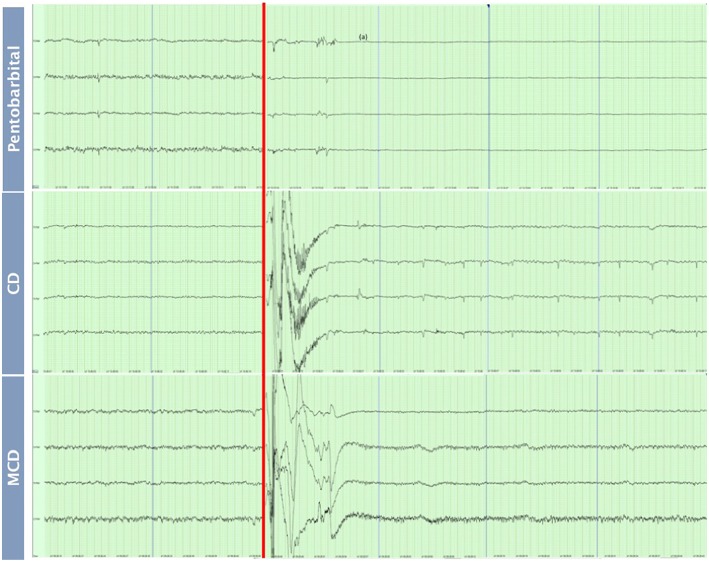
Raw EEG recordings of baseline (30 s) and after euthanasia methods application of pentobarbital sodium, manual cervical dislocation (CD), mechanical cervical dislocation (MCD) in chickens (first 60 s after euthanasia method application). Red line: Application of euthanasia method. Blue lines: end of a 15 s interval. (a) Isoelectric EEG.

**Figure 3:**
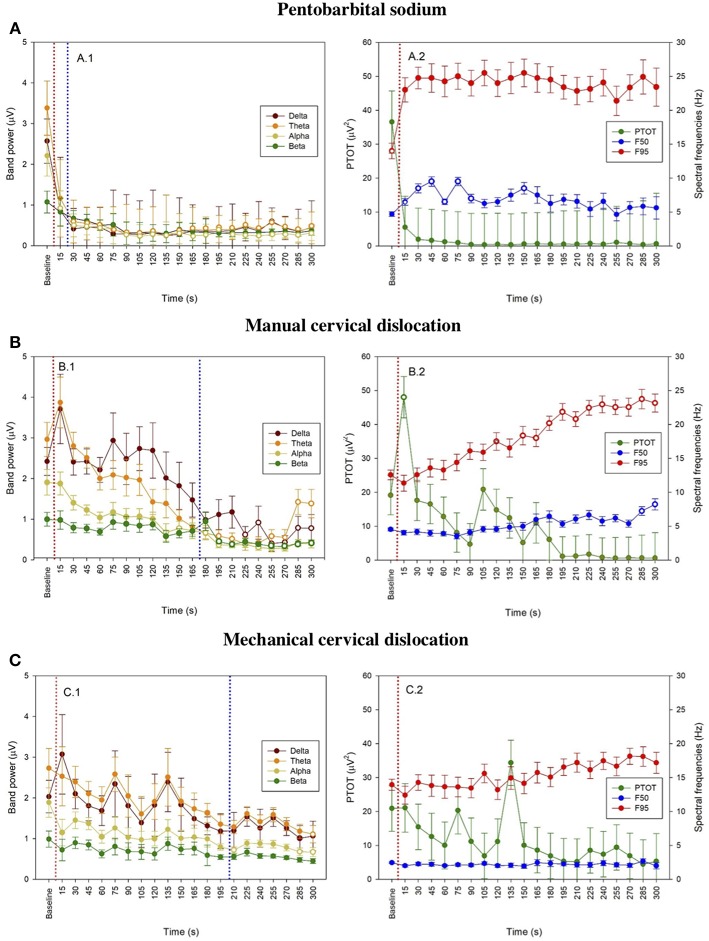
Time series for pentobarbital sodium **(A)**, manual cervical dislocation **(B)**, mechanical cervical dislocation **(C)** of mean frequency band powers (±SE) (A.1, B.1, C.1), mean (±SE) PTOT and mean spectral frequencies (±SE) (i.e., F50 and F95) (A.2, B.2, C.2) from baseline to 5 min endpoint (300 s) in laying hens. Data is shown for consecutive 15 s intervals after euthanasia application. Baseline values represent the mean of four 15 s intervals immediately prior euthanasia. Open symbols indicate that the time points differed significantly compared to baseline (adjusted *p* < 0.05). The dotted red line represents time of euthanasia method application and dotted blue line represents first time of isoelectric EEG.

The pattern of changes in EEG activity differed according to the treatment (Figures 2, 3). There was an increase of artifact movement affecting the signal immediately after cervical dislocation methods, as observed in the raw EEG (Figure 2). QEEG analyses showed a prevalence of low frequency bands (theta and delta frequency bands) with an increased Ptot and decreased F50 and F95 values, reflecting the birds' loss of sensibility during baseline. After barbiturate administration, all birds showed a sustained decrease in all mean frequency bands and Ptot with a significant increase in mean F50 and F95 starting at 15 s post-injection (Table 8). Mean alpha frequency power decreased significantly at 15 s (adjusted *p* = 0.04) and was followed by significant differences in mean theta and delta bands at 30 and 45 s post-injection, respectively (adjusted *p* = 0.03; adjusted *p* = 0.03, respectively), compared with baseline. Significant increases in mean F50 and mean F95 (adjusted *p* = 0.0006; adjusted *p* = 0.002, respectively) were observed at 15 s after administration. All birds presented isoelectric EEGs with a mean time to onset of isoelectric EEG at 20 ± 23 s; significantly different (*p* < 0.0001) compared with CD and MCD methods (171 ± 15 s and 207 ± 23 s, respectively) (Figure 2 and Table 4). For CD, there was an increase in mean F95 from 120 to 300 s, with a decrease in mean alpha from 135 to 300 s, compared to baseline (adjusted *p* ≤ 0.05, Table 9). For mean delta, theta and beta bands, CD technique demonstrated a significant decrease, relative to baseline from 180 to 300 s (adjusted *p* ≤ 0.05). In addition, mean F50 was higher from 285 to 300 s, compared to baseline (adjusted *p* = 0.04). For MCD, only mean alpha and theta showed significant differences, compared to mean baseline time points, at 285 and 300 s, respectively (adjusted *p* < 0.05) (Table 10). No other EEG variables differed significantly from baseline in chickens on-farm killed by MCD. Similar to CD, mean F95 values tendended to higher values toward the end of the recording after MCD (adjusted *p* < 0.05). There was an increase in mean Ptot (adjusted *p* = 0.005) and mean delta and theta (adjusted *p* > 0.05) at 15 s after CD application, while for MCD a high peak was observed at 135 s (adjusted *p* > 0.05).

**Table 9:** Mean EEG parameters (SE) after manual cervical dislocation.

**Time (s)**	**Delta (μV)**	**Theta (μV)**	**Alpha (μV)**	**Beta (μV)**	**F50 (Hz)**	**F95 (Hz)**	**PTOT (μV^**2**^)**	N epochs^**^
	**Mean (SE)**	**Mean (SE)**	**Mean (SE)**	**Mean (SE)**	**Mean (SE)**	**Mean (SE)**	**Mean (SE)**	
Baseline	2.4 (0.4)	3.0 (0.4)	1.9 (0.3)	1.0 (0.2)	4.5 (0.2)	12.6 (0.7)	19.2 (5.8)	80
15	3.7 (0.9)	3.9 (0.6)	1.9 (0.3)	1.0 (0.2)	4.1 (0.3)	11.3 (1.2)	48.0 (6.1)*	18
30	2.4 (0.3)	2.8 (0.3)	1.4 (0.2)	0.8 (0.1)	4.2 (0.4)	12.6 (1.0)	17.6 (6.0)	19
45	2.4 (0.3)	2.5 (0.3)	1.2 (.1)	0.8 (0.1)	4.0 (0.4)	13.6 (1.3)	16.5 (5.8)	20
60	2.2 (0.3)	2.0 (0.3)	1.0 (0.1)	0.7 (0.1)	3.9 (0.3)	13.3 (1.4)	12.9 (5.8)	20
75	2.9 (0.7)	2.1 (0.4)	1.2 (0.2)	0.9 (0.2)	3.5 (0.4)	14.4 (1.2)	8.1 (5.8)	20
90	2.5 (0.6)	2.0 (0.5)	1.1 (0.2)	0.9 (0.2)	4.1 (0.4)	16.1 (1.2)	4.7 (6.1)	18
105	2.7 (0.5)	2.0 (0.4)	1.1 (0.2)	0.8 (0.2)	4.6 (0.4)	15.9 (1.2)	20.9 (6.1)	18
120	2.7 (0.7)	1.4 (0.3)	1.0 (0.2)	0.9 (0.1)	4.6 (0.4)	17.5 (1.3)*	14.8 (6.1)	18
135	2.0 (0.6)	1.4 (0.5)	0.7 (0.2)*	0.6 (0.1)	4.9 (0.6)	16.5 (1.3)	12.4 (6.0)	19
150	1.8 (0.6)	1.0 (0.2)	0.8 (0.2)*	0.7 (0.1)	5.0 (0.6)	18.4 (1.4)*	5.2 (6.0)	19
165	1.5 (0.4)	0.8 (0.2)	0.7 (0.2)*	0.7 (0.1)	6.0 (0.8)	18.0 (1.3)*	11.0 (6.0)	19
180	1.0 (0.2)*	0.7 (0.2)*	0.7 (0.2)*	0.9 (0.2)	6.5 (0.8)	20.2 (1.2)*	6.1 (6.1)	18
195	1.1 (0.4)	0.6 (0.2)*	0.4 (0.1)*	0.4 (0.1)*	5.3 (0.5)	21.9 (1.2)*	1.1 (5.8)	20
210	1.2 (0.4)	0.5 (0.2)*	0.4 (0.1)*	0.4 (0.1)*	6.1 (0.6)	20.8 (1.1)*	1.2 (6.0)	19
225	0.6 (0.2)	0.4 (0.2)*	0.4 (0.1)*	0.4 (0.1)	6.7 (0.7)	22.5 (1.2)*	1.7 (6.0)	20
240	0.9 (0.4)	0.4 (0.2)*	0.3 (0.1)*	0.4 (0.1)*	5.8 (0.7)	23.0 (1.2)*	0.8 (6.0)	20
255	0.4 (0.2)*	0.6 (0.2)*	0.3 (0.1)*	0.3 (0.0)*	6.2 (0.5)	22.5 (1.2)*	0.6 (6.1)	18
270	0.4 (0.2)*	0.6 (0.2)*	0.3 (0.1)*	0.3 (0.0)*	5.4 (0.5)	22.6 (1.3)*	0.7 (6.1)	18
285	0.8 (0.3)*	1.4 (0.3)*	0.4 (0.1)*	0.4 (0.1)*	7.2 (0.6)*	23.7 (1.4)*	0.7 (6.7)	15
300	0.8 (0.3)*	1.4 (0.4)*	0.4 (0.1)*	0.4 (0.1)*	8.2 (0.8)*	23.2 (1.3)*	0.6 (7.5)	12

*Comparison of the mean and standard error (SE) of the frequency band powers (delta, theta, alpha, beta), spectral frequencies [median frequency (F50) and spectral edge frequency (F95)] and absolute power (PTOT) of the avian electroencephalogram at consecutive 15 s intervals after applying manual cervical dislocation method (CD) in laying hens. Data are shown as mean values over consecutive 15 s intervals after euthanasia, with baseline representing the mean of the 60 s immediately euthanasia. Asterisks indicate values within columns that are significantly different from baseline (adjusted p ≤ 0.05)*.

** *Number of analyzed epochs varies as not every epoch per 15 s interval was available for every bird. Baseline parameter are based on 4 epochs per bird, post-euthanasia parameters are based on 20 epochs obtained in a 5 min recording*.

**Table 10:** Mean EEG parameters (SE) after mechanical cervical dislocation.

**Time (s)**	**Delta (μV)**	**Theta (μV)**	**Alpha (μV)**	**Beta (μV)**	**F50 (Hz)**	**F95 (Hz)**	**PTOT (μV^**2**^)**	N epochs^**^
	**Mean (SE)**	**Mean (SE)**	**Mean (SE)**	**Mean (SE)**	**Mean (SE)**	**Mean (SE)**	**Mean (SE)**	
Baseline	2.0 (0.4)	2.7 (0.5)	1.9 (0.4)	1.0 (0.2)	4.9 (0.2)	13.9 (0.8)	20.9 (6.7)	60
15	3.1 (1.0)	2.5 (0.7)	1.2 (0.3)	0.7 (0.3)	4.0 (0.4)	12.4 (1.3)	21.1 (7.2)	14
30	2.1 (0.4)	2.4 (0.4)	1.5 (0.2)	0.9 (0.2)	4.5 (0.5)	14.3 (1.2)	15.5 (6.7)	15
45	1.8 (0.4)	2.1 (0.3)	1.4 (0.2)	0.9 (0.1)	4.4 (0.5)	13.8 (1.6)	12.6 (6.9)	14
60	1.7 (0.4)	2.0 (0.3)	1.1 (0.2)	0.6 (0.1)	4.0 (0.3)	13.7 (1.7)	10.0 (6.9)	14
75	2.3 (0.8)	2.6 (0.4)	1.3 (0.3)	0.8 (0.2)	4.3 (0.5)	13.6 (1.5)	20.3 (6.9)	14
90	1.8 (0.7)	2.1 (0.5)	1.0 (0.2)	0.7 (0.2)	4.2 (0.5)	13.4 (1.4)	11.2 (6.9)	14
105	1.4 (0.6)	1.6 (0.4)	1.0 (0.2)	0.7 (0.2)	4.7 (0.5)	15.6 (1.4)	6.9 (6.7)	15
120	1.8 (0.8)	1.9 (0.4)	1.0 (0.2)	0.6 (0.2)	4.0 (0.5)	13.2 (1.5)	11.1 (6.7)	15
135	2.4 (0.7)	2.5 (0.7)	1.2 (0.2)	0.9 (0.2)	4.1 (0.7)	14.9 (1.7)	34.3 (6.7)	15
150	1.8 (0.6)	1.9 (0.4)	1.0 (0.2)	0.7 (0.1)	3.9 (0.6)	14.2 (1.5)	10.0 (6.7)	15
165	1.5 (0.5)	1.7 (0.4)	1.0 (0.2)	0.8 (0.2)	4.9 (0.9)	15.7 (1.5)	8.6 (6.7)	15
180	1.3 (0.2)	1.6 (0.2)	1.0 (0.2)	0.6 (0.3)	4.7 (0.9)	15.1 (1.4)	6.9 (6.7)	15
195	1.2 (0.4)	1.4 (0.2)	0.8 (0.2)	0.6 (0.1)	4.5 (0.6)	16.5 (1.2)	5.3 (6.7)	15
210	1.2 (0.5)	1.3 (0.2)	0.7 (0.2)	0.6 (0.1)	4.3 (0.7)	17.2 (1.4)	5.2 (6.7)	15
225	1.5 (0.2)	1.6 (0.2)	0.9 (0.2)	0.7 (0.1)	4.3 (0.8)	16.1 (1.3)	8.5 (6.7)	15
240	1.3 (0.5)	1.4 (0.2)	0.9 (0.1)	0.6 (0.1)	4.8 (0.8)	17.5 (1.3)	7.4 (6.7)	15
255	1.5 (0.2)	1.6 (0.2)	0.9 (0.1)	0.6 (0.1)	4.3 (0.6)	16.7 (1.4)	9.4 (6.7)	15
270	1.3 (0.2)	1.4 (0.2)	0.8 (0.1)	0.6 (0.04)	4.1 (0.6)	18.1 (1.2)	6.9 (6.7)	15
285	1.0 (0.3)	1.2 (0.2)	0.7 (0.1)*	0.5 (0.1)	5.4 (0.7)	18.1 (1.4)	4.5 (7.2)	13
300	1.1 (0.4)	1.1 (0.2)*	0.7 (0.1)*	0.5 (0.1)	4.0 (0.9)	17.2 (1.6)	5.3 (8.2)	10

*Comparison of the mean and standard error (SE) of the frequency band powers (delta, theta, alpha, beta), spectral frequencies [median frequency (F50) and spectral edge frequency (F95)] and absolute power (PTOT) of the avian electroencephalogram at consecutive 15 s intervals after applying mechanical cervical dislocation method (MCD) in laying hens. Data are shown as mean values over consecutive 15 s intervals after euthanasia, with baseline representing the mean of the 60 s immediately euthanasia. Asterisks indicate values within columns that are significantly different from baseline (adjusted p ≤ 0.05)*.

** *Number of analyzed epochs varies as not every epoch per 15 s interval was available for every bird. Baseline parameter are based on 4 epochs per bird, post-euthanasia parameters are based on 20 epochs obtained in a 5 min recording*.

There was a significant body weight × method interaction observed in delta, theta, alpha, and beta (Table 6) and changes in slopes and contrasts of frequency bands compared to body weight (Table 11). There was a significant non-linear relationship with body weight effect for Ptot (body weight × body weight, *p* < 0.001). MCD has significant positive slopes in all frequency bands, while CD had significant negative slopes with all bands. Pentobarbital sodium method had no significant changes in slope values, suggesting no body weight effect on the administered dose. Contrasts of the slopes showed MCD to be significantly more positive than CD in delta, theta, alpha, and beta (*p* = 0.01, *p* =< 0.0001, *p* = 0.003, *p* < 0.0001; respectively). MCD was significantly more negative than pentobarbital sodium in delta and beta (*p* = 0.03 and *p* = 0.06; respectively). Conversely, the contrast of the slops showed that MCD was not significantly different from pentobarbital sodium in delta, alpha, and beta (*p* = 0.4, *p* = 0.07, *p* = 0.06; respectively). For MCD vs. pentobarbital, there was a significant contrast (*p* = 0.02). The slope of theta × body weight was significantly more positive in the MCD method compared to pentobarbital sodium (*p* = 0.02). The contrasts of the slopes of CD and pentobarbital sodium revealed no significant differences in alpha, theta, and beta (*p* = 0.07, *p* = 0.4, *p* = 0.06, respectively), but were significant in delta (*p* = 0.03).

**Table 11:** Summary effects of euthanasia method and body weight interaction on EEG frequency bands in hens.

**Euthanasia method**	**Delta**		**Theta**		**Alpha**		**Beta**	
	**Slope**	***p-*value**	**Slope**	***p-*value**	**Slope**	***p-*value**	**Slope**	***p-*value**
MCD	2.1	0.01^*^	2.6	<0.0001^*^	1.06	0.007^*^	0.6	0.0005^*^
CD	−1.8	0.053	−1.0	0.12	−0.6	0.07	−0.6	0.001
Pentobarbital sodium	1.0	0.3	0.5	0.4	0.2	0.5	0.1	0.5

* *Indicate values that are significantly different (p < 0.05)*.

### Relationship Between EEG and Insensibility Parameters

**Figure 4:**
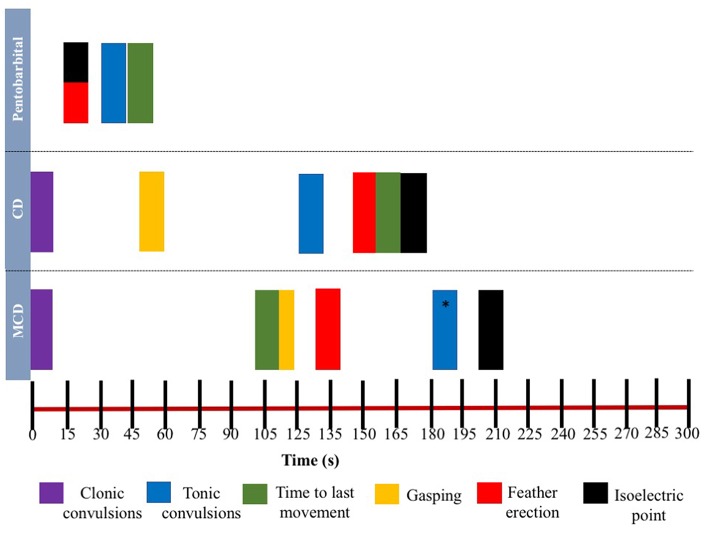
Relationship between time and mean latencies for key behavioral events (gasping, feather erection, clonic, and tonic convulsions and time to last movement) as well as mean isoelectric point in EEG during 5-min post-application of euthanasia method in laying hens. *Tonic convulsions were only observed in Smoky Joe strain group.

The relationship between latencies of behavioral responses and EEG changes for the three euthanasia and on-killing methods are illustrated in Figure 4. Pentobarbital sodium administration resulted in a rapid decrease in overall EEG activity with isoelectric EEG in all birds and occasionally followed by tonic convulsions (Table 3). In contrast, birds killed with physical methods presented with clonic-tonic convulsions prior to visualization of isoelectric points in the raw EEG. An initial sharp increase in Ptot (adjusted *p* = 0.005) at 15 s was noted in CD treatment, which occurred approximately at the same time as the onset of clonic convulsions (2 ± 0.2 s). A second increase in Ptot was noted from 105 to 135 s after CD application and occurred approximately at the same time as the onset of the tonic convulsions (130 ± 20 s). However, changes in mean Ptot did not differ from baseline at time point 15 s in birds undergoing MCD on-farm killing method, despite of exhibiting clonic convulsions approximately at this time point. In addition, the sharp increase in Ptot observed at 135 s after MCD application was not associated with a relevant behavioral or physiological response. Time to the last movement was observed before the onset of isoelectric EEG after CD and MCD, but not in pentobarbital sodium birds. There were non-significant relations between time to last movement and isoelectric EEG after pentobarbital sodium, CD, or MCD application (*p* = 0.89; *p* = 0.2; *p* = 0.6, respectively). Latency to feather erection and gasping likely did not cause artifact noise in the EEG and could not be associated with a specific EEG change.

## Discussion

This study identified significant differences between CD, MCD, and pentobarbital sodium for the onset of brain death based on changes in EEG and latencies to behavioral and physiological responses. Brain death was estimated from EEG changes by comparing baseline values and onset of isoelectric EEG in isoflurane-anesthetized hens. Behavioral and physiological changes, including convulsions, gasping, feather erection, and time to the last movement, were analyzed for correlation with isoelectric EEG changes. MCD using the KED (model C) resulted in a longer time to brain death compared to CD and pentobarbital sodium. Notably, only 8 of 15 hens on-farm killed with this device presented isoelectric EEG around 207 s, while the other birds did not have EEG changes associated with brain death before the 5-min endpoint. These 7 hens did not show signs of distress or humane endpoint events that required a second euthanasia method application, suggesting a potentially higher failure rate and indicating welfare concerns for the MCD method compared to CD and anesthetic overdose methods. Manual cervical dislocation was the most reliable physical method since all birds presented signs of brain death based on EEG changes. Pentobarbital sodium caused the shortest time to brain death compared to cervical dislocation methods, and a consistent isoelectric EEG pattern in all birds euthanized with this method. The IV pentobarbital sodium overdose method was confirmed to be a more humane method for adult laying hens by causing rapid onset to brain death based on EEG, behavioral, and physiological findings.

Previous research has described an association between cessation of movement and electroencephalographic inactivity to determine brain death (38, 39). However, differences in euthanasia or killing methods, species, strain, age, data collection, and analysis among the previous studies do not allow direct comparison. Using latency to isoelectric EEG as an indicator of brain death (27), there was no significant interaction, but there was a pattern of cessation of movement followed by isoelectric EEG with the cervical dislocation methods. Some of the birds euthanized with pentobarbital sodium showed a different pattern where the onset of brain death was followed by the time to last movement, which has been reported in humans (40). A limitation of using the isoelectric EEG visually scored from the raw EEG tracings as an indicator of brain death is that its interpretation could have been affected by extracranial noise during euthanasia and hence presented a delayed onset compared to the time to last movement with the cervical dislocation methods. Regardless, in terms of brain death, quantitative EEG (QEEG) results confirm there is no rapid latency to brain death after MCD as it occurs at ~285 s based on increased F50 and F95 values (3, 19) and decreases in Ptot, delta, and theta patterns (19, 39, 41). Manual cervical dislocation significantly reduced some EEG measures between ~120–135 s with isoelectric EEG values observed in all birds at 171 s after application. Significant QEEG changes occurred between 15 and 30 s after pentobarbital administration with an observed isoelectric EEG at ~20 s. The reported patterns of QEEG changes are similar to previous reports in chickens, in which methods involving the stretching of the neck produced a more rapid change in the EEG measures compared with methods involving a tool or crushing of the vertebrae (3, 8). This study is one of the few that includes different methods of quantitative EEG analysis as a conservative measure to evaluate brain death in chickens. Spectral frequencies analysis and frequency bands analysis showed similar results on time to brain death across treatments. The onset of isoelectric EEG based on visual scoring in the raw EEG occurred approximately at the same time as the quantifiable changes in the EEG spectrum for pentobarbital sodium and CD. For MCD, the differences observed in the timing of changes between isoelectric values and spectrum changes are possible because the birds presenting isoelectric EEG were not removed from the time series or the presentation of convulsions affected the EEG signal.

Manual and mechanical cervical dislocations (MCDs) were relatively easy to apply in all birds, although CD and MCD were affected by body weight, as the power of the EEG frequency bands were affected by the body weight. For CD, there was an overall decrease of EEG power on the frequency bands as body weight increased (negative slope) suggesting a better method efficiency with heavier birds. This seems to be primarily due to the operator's ability to adapt to the individual bird variation in weight (3) with heavier birds being easier to restrain. In contrast, the positive slope in MCD suggests that as the bird's weight increased the EEG power was less likely to decrease. Unlike CD, the MCD does not allow the operator to modify the technique according to the bird's individual body weight variation (5). Body weight had no effect on the pentobarbital sodium method (other than dose). These findings concur with reported brain death patterns and incidence of isoelectric EEG seen across treatments. The non-linear relationship with body weight and Ptot suggest that as body weight increases or decreases to a point there is no effect of the body weight and the euthanasia or killing methods used on the Ptot values.

There was a limited association between the EEG responses and other physiological and behavioral parameters, as described by Martin et al. (3). Feather erection, gasping, and tonic and clonic convulsions were not observed in all birds across methods. Similar results have been described in previous studies that indicate some behaviors are not method-specific (3, 7, 21). The differences in behavioral responses and EEG changes between treatments are likely secondary to anatomical damage caused by the methods. Manual cervical dislocation causes anatomical damage by the stretching of the neck that results in a greater degree of subdural and parenchymal hemorrhage compared to MCD (5). Although gross scoring of the lesions was not attempted in this study, EEG changes in frequency patterns and shorter latency to isoelectric EEG confirm severe brain trauma in CD compared to MCD in chickens. Gross dissections were not conducted because some birds received a secondary euthanasia method to confirm death via a non-penetrating captive bolt, which alters head and neck anatomy (20, 37). Both cervical dislocation methods are supposed to transect the spinal cord with more cervical damage caused by MCD than CD (5). This trauma to spinal cord and neck muscles has been suggested to cause hypoxia and cerebral ischemia with a reflex response observed as gasping (5). In this study, CD and MCD caused gasping (5, 21). However, a shorter latency to gasping was observed after CD compared to MCD. It is unclear if this response is biologically relevant or significant in terms of method efficacy. Similarly, Cors et al. (42) considered gasping to be a reflex response not necessarily related to a respiratory movement based on EEG findings in turkeys. The few reported numbers of chickens not exhibiting gasping after cervical dislocation was because the bird's beak was out of view from the camera and not necessarily absent. Clonic and tonic convulsions are not useful indicators of brain activity in poultry (3, 42). The convulsions are neuromuscular spasms likely involuntary, secondary to brain anoxia (16) or metabolic encephalopathies (40), and are commonly observed after stunning with physical methods in chickens and turkeys (7, 12, 43). The latency to clonic convulsions was likely not affected by the anesthesia model since a rapid onset of convulsions is reported in chickens and turkeys after application of cervical dislocation and non-penetrating captive bolt devices (3, 12, 15, 20). Woolcott et al. (21) reported that the presentation of clonic convulsions could be affected by anesthesia drugs, and the authors do not recommend using clonic convulsions for assessing brain death in anesthetized turkeys. However, there was not a clear pattern or transition from clonic to tonic convulsions, and tonic convulsions were absent after MCD in white Leghorn hens. It could be that in those birds, the response was going to occur after the 5-min end-point. Feather erection has been suggested to be an indicator of cardiac arrest (7, 44), but its presentation does not have a clear association with brain death. In this study, cardiac function could not be consistently assessed due to clonic-tonic convulsions and restraint limitations, but recent investigations with turkeys confirm no association between feather erection and cardiac function (34) and evidence of a heartbeat (not necessarily associated with a functional heart waveform) after the onset of brain death (21). In addition, a limitation of the behavioral analysis of the CD application compared to pentobarbital sodium and MCD methods was the short time delay from CD application to placement of the birds on the table for assessment. This delay on the initial observation after CD application was also described in previous work with chickens (15). Similar to Jacobs et al. (15) findings on efficiency of the KED vs. CD in broiler chickens, the CD method consistently demonstrated a shorter duration of convulsions or latency to the cessation of movements suggesting the CD method performs better than the MCD or KED in chicken (15). Another limitation of the study design was the order in which each strain was tested on different days such that the strain order could not be randomized. The study was not originally designed to examine a strain effect, but it was controlled for by including it in the model. The strain effect interactions were not significant in the behavioral analysis. More conservative indicators of insensibility, such as eye reflexes, could not be measured due to method (the face, including the eyes, remained within the facemask in most birds and data was not collected if convulsions occurred at the same time) and strain (Smoky Joe strain hens have inherited ocular development that impedes pupillary eye reflex assessment) limitations (45).

From an animal welfare standpoint, time to insensibility is more relevant than time to brain death. However, a minimal anesthesia model was used to preserve the bird welfare given the welfare concerns with the MCD method as described in previous research in chickens (5, 15). Efforts were made to limit the anesthetic effects (use of inhaled vs. injectable agents, rapid induction and electrode placement, and minimal maintenance levels during baseline EEG recordings), and to minimize inter-operator bias, with the same operator performing euthanasia or killing techniques throughout. Minimal anesthesia models with gas anesthetics have been validated in veterinary research where the welfare of the animal is at risk (46–48). Martin et al. (33) calculated a threshold of F50 <6.8 for determining insensibility in broiler chickens. In this study, all hens exhibited mean F50 Hz <6.8 Hz from baseline to time to isoelectric EEG, if present. This suggests that the birds were insensible during the time of method application until brain death. Values >6.8 Hz in F50 are likely secondary to extra cranial noise that contaminated the F50 frequency or EEG signal (34). In addition, insensibility from baseline (i.e., the prevalence of theta and delta brain activity) until brain death (decrease in delta and theta mean values compared to baseline) (39). The acute increases in Ptot observed after CD and MCD application were interpreted as an overall increase in the EEG activity and not necessarily a response to noxious stimulation, as suggested by previous studies on decapitation in rats (46, 49). Martin et al. (3), described similar increases in Ptot after application of different mechanical and manual dislocation techniques in laying hens and broilers lightly anesthetized with sevoflurane. Martin et al. interpreted these changes as resulting from heightened cerebrocortical activity or convulsions that could indicate loss of cerebrocortical function (3). Some authors also suggest that these changes could be secondary to severe ischemia, hypoglycemia or electrolyte metabolic disorders as reported in human encephalographic studies (50–52). Changes in the EEG related to ischemia are the result of a loss of neuronal transmembrane gradients observed as an increase in slower frequencies (delta and theta) (50).

This study assessed the onset of brain death in hens when on-farm killed by either mechanical cervical dislocation using a commercial tool (Koechner Euthanizing Device, model C) or manual cervical dislocation compared to IV pentobarbital sodium euthanasia. Both cervical dislocation methods are demonstrated to transect the spinal cord (5); however, the MCD, using KED-C, was shown to be inefficient by presenting unpredictable physiological, behavioral, and EEG responses compared with CD and pentobarbital sodium. This highlights the welfare concerns of using forceps or pliers as a tool for assisted cervical dislocation in chickens (8, 9, 11). The use of CD still has several welfare concerns, including the prolonged time to brain death, potential incorrect site of dislocation, and variability in operator skill, technique, and strength (3, 4, 8).

The mechanical cervical dislocation method was inconsistent in causing brain death compared to CD and pentobarbital sodium based on EEG evaluation and physiological and behavioral findings. The onset of brain death occurred sooner after IV pentobarbital sodium and manual cervical dislocation than after mechanical cervical dislocation. IV pentobarbital sodium is a preferred euthanasia method for adult laying hens, but it is not practical to use on-farm and requires administration by a licensed veterinarian (1, 2). This study provides quantitative data for future welfare recommendation on appropriate on-farm laying hen euthanasia and killing methods.

## Data Availability

The datasets used and analyzed during the current study are available from the corresponding author on request.

## Ethics Statement

All animals used and procedures were approved by the University of Guelph Animal Care Committee (eAUP 3321). The institution is registered under the Animals for Research Act of Ontario and holds a Good Animal Practice certificate issued by the Canadian Council on Animal Care.

## Author Contributions

TW, ST, PT, and KS-L conceived of the work and prepared the grants. TW, ST, PT, FJ, and EH jointly designed the study. EH, FJ, and PT conducted the work. GM and EH conducted the statistical analyses and all authors reviewed the results. EH and PT prepared the manuscript. All authors reviewed and approved the final manuscript.

### Conflict of Interest Statement

TW is the Egg Farmers of Canada Chair in Poultry Welfare Research. The remaining authors declare that the research was conducted in the absence of any commercial or financial relationships that could be construed as a potential conflict of interest.
