# Supplementary material for: Evaluation of Brain Death in Laying Hens During On-Farm Killing by Cervical Dislocation Methods or Pentobarbital Sodium Injection
Source: Front Vet Sci. 2019 Sep 3;6:297. doi: 10.3389/fvets.2019.00297 (PMC6733910; doi:10.3389/fvets.2019.00297)
Supplement: Supplementary file 1 [file Data_Sheet_1.docx]

**Supplementary tables**

**Supplementary Table 1.** Full summary effects of euthanasia method on EEG frequency bands and Ptot in white Leghorn and Smoky Joe hens*

|  | **Delta** | | **Theta** | | **Alpha** | | **Beta** | | **PTOT** | |
| --- | --- | --- | --- | --- | --- | --- | --- | --- | --- | --- |
| Effect | *F* | Pr >*F* | *F* | Pr >*F* | *F* | Pr >*F* | *F* | Pr >*F* | *F* | Pr >*F* |
| Method | 1.6 | 0.2 | 7 | 0.001 | 0.7 | 0.5 | 8.46 | 0.0003 | 0.7 | 0.5 |
| Time | 2.8 | <0.0001 | 6.6 | <0.0001 | 3.3 | <0.0001 | 5.2 | <0.0001 | 3.3 | <0.0001 |
| Strain | 0.9 | 0.4 | 0.7 | 0.4 | 0.5 | 0.5 | 0.08 | 0.8 | 0.5 | 0.5 |
| Body weight | 0.8 | 0.4 | 0.4 | 0.5 | 1.7 | 0.2 | 0.1 | 0.8 | 1.7 | 0.2 |
| Strain x time | 1.6 | 0.2 | 2.8 | 0.0001 | 3.3 | <0.0001 | 2.8 | 0.0001 | 2.4 | 0.0007 |
| Body weight x Method | 5.1 | 0.006 | 6.9 | 0.001 | 4.7 | 0.001 | 11.66 | <0.0001 | 0.6 | 0.6 |

*Only significant interactions are included

**Supplementary Table 2.** Full summary effects of euthanasia method on EEG spectral frequencies in white Leghorn and Smoky Joe hens*

|  | F50 | | F95 | |
| --- | --- | --- | --- | --- |
| Effect | *F* | Pr >*F* | *F* | Pr >*F* |
| Method | 0.3 | 0.7 | 0.8 | <0.0001 |
| Time | 2.4 | 0.001 | 2.8 | <0.0001 |
| Strain | 0.3 | 0.6 | 0.1 | 0.7 |
| Body weight | 0.8 | 0.4 | 0.9 | 0.3 |
| Strain x treatment | 3.9 | 0.02 | 5.4 | 0.006 |
| Strain x time x method | 2.6 | <0.0001 | 0.8 | 0.8 |

*Only significant interactions are include
